# Valeric acid acts as a novel HDAC3 inhibitor against prostate cancer

**DOI:** 10.1007/s12032-022-01814-9

**Published:** 2022-09-29

**Authors:** Rui Han, Hongxing Yang, Ya Li, Changquan Ling, Lingeng Lu

**Affiliations:** 1 Department of Chinese Medicine, Naval Medical University, Shanghai, 200433 People’s Republic of China; 2 Department of Chinese Medicine Oncology, Changhai Hospital affiliated to Naval Medical University, Shanghai, 200433 People’s Republic of China; 3 Beijing University of Traditional Chinese Medicine, Beijing, 100700 People’s Republic of China; 4 Department of Integrated Chinese and Western Medicine & Geriatric Oncology, Peking University Cancer Hospital, Beijing, 100142 People’s Republic of China; 5 Department of Oncology, The First Affiliated Hospital of Guangzhou University of Chinese Medicine, Guangzhou, 510405 Guangdong People’s Republic of China; 6 Present Address: Department of Chronic Disease Epidemiology, Yale School of Public Health, Yale University, 60 College Street, New Haven, CT 06520-8034 USA; 7 School of Medicine, Center for Biomedical Data Science, Yale University, 60 College Street, New Haven, CT 06520-8034 USA; 8 Yale Cancer Center, Yale University, 60 College Street, New Haven, CT 06520-8034 USA

**Keywords:** Valeric acid, HDAC inhibitor, Prostate cancer, Cancer stemness, Apoptosis

## Abstract

Prostate cancer is the second cause of cancer-related deaths in men worldwide, and new agents for curing the disease are still needed. In this study, we theoretically and experimentally demonstrated that valeric acid (VA) was a HDAC inhibitor, and anti-cancer efficacy of VA in prostate cancer cells was also observed using either 2D or 3D culture systems. VA was cytotoxic for prostate cancer cells but low toxic to normal cells. VA significantly inhibited *E2F1*/*E2F3* expression but increased CASP3 activity. In vivo mouse models further showed its anti-cancer activity and potential property of chemosensitizer with promoting apoptosis. The findings suggest that VA acts as a HDAC3 inhibitor with anti-cancer effect on prostate cancer by regulating *E2F1*/*E2F3*/CASP3 axis.

## Introduction

Prostate cancer (PCa) is one of the most common cancer types in men with over 200,000 mortality each year worldwide [1], which ranks the second in all male cancer-caused deaths [2]. Due to its occult onset, most PCa patients have been at stage III or IV when diagnosed. For patients who have already lost the chance of surgical resection, the treatment options are limited, such as chemotherapy, radiotherapy, and endocrinotherapy [3], due to their limited clinical benefits to the patients. Thus, it is still unmet to develop novel therapies for the disease.

Histone deacetylase (HDAC) is a key enzyme in controlling chromatin modification and regulating important cellular processes, such as cell cycle progression and apoptosis [4]. HDACs can remove the acetyl group from histone proteins, making chromatin less accessible to transcriptional factors and consequently mediating transcriptional regulation and post-translational modification [5]. HDACs are overexpressed in many human cancers, including prostate cancer, and the overexpression of HDAC3 has been found in a positive correlation with the proliferation, development, and poor prognosis of prostate cancer [6]. The over-action of HDACs on the histone and non-histone substrates can lead to reduced expression of tumor suppressor or dysregulate the signal pathways of cancer by modifying key molecules [7]. These previous findings suggest that inhibiting HDACs may have potential antitumor effect. Thus far, several HDAC inhibitors (HDACi) have been in the pipeline for the investigation of cancer treatment [8, 9].

Valerian (Valeriana officinalis) is an herb, which has been used for treating urinary diseases in Chinese traditional medicine for thousands of years. Our previous studies has found that Valeric acid, as a major active component of Valerian, was a potential HDAC inhibitor which could strongly increase the apoptosis with anti-cancer effects on liver and breast cancer [10, 11]. In this study, we aimed to further dig the potential of VA against prostate cancer. Swiss Target Prediction assay combined with Network pharmacology assay were applied to predict the potential target of VA. Anti-HDAC and anti-proliferative abilities assays have been determined using prostate cancer in vitro and in vivo.

## Materials and methods

### Swiss target prediction assay

Potential molecule target prediction of VA was conducted using Swiss Target Prediction tool based on 2D and 3D structure of VA (www.swisstargetprediction.ch). All parameters were set as default.

### Network pharmacology assays

We searched the potential effective targets of Valeric Acid using the PharmMapper database (http://www.lilab-ecust.cn/pharmmapper, version 2017), performed Protein–Protein Interaction (PPI) analysis using STRING database (http://string-db.org/cgi/input.pl?sessionId=foKarBDd2Huz&input_page_show_search=on; version 11.0), and visualized the pathways by a software of Cytoscape (version 3.7.0, https://cytoscape.org/). Gene Ontology (GO) function and KEGG (Kyoto Encyclopedia of genes and genomes) pathway analyses were carried out by R packages (version 3.6.1) software.

### Cell culture

The prostate cancer cell lines PC‐3 and DU145 and prostate normal cell lines RWPE-1 and RWPE-2 were obtained from ATCC (USA) in August 2021 and identified by the STR assay. Both cancer cells were cultured in either ATCC-formulated F-12 K Medium or EMEM with 10% FBS, respectively. Two normal cell lines were cultured in completed Keratinocyte Serum-Free Medium (Cat No.17005–042) (Invitrogen, USA). All cells were maintained in a 37 °C-humidified incubator supplied with 5% CO_2_.

For establishing HDAC3-knockout clones, PC-3 and DU145 cells were both conducted using HDAC3 human gene-knockout kit (Crispr) (OriGene Technologies, USA) following the manufacturer’s instruction as described in our previous study [12]. Briefly, prior to the transfection, 3 × 10^5^ PC-3 or DU145 cells were grown in 2-mL culture media in each well of a 6-well plate for 24 h. Three transfections were then set up in complete medium. Two days after the transfection, 1 μg/mL puromycin selection (5 days) were performed for obtaining individual cell colonies. PCR were then applied to verify the knockout of the interest gene.

### MTS cell proliferation assay

MTS cell proliferation assay was used to determine the anti-cancer ability of VA for selected cell lines. The final concentration of 100 µM VA was selected as a working concentration based on our previous study [10]. The assay was performed by adding 20 μL of MTS solution (Promega, USA) into each well in a dark hood at different incubation time points (24 h, 48 h, and 72 h). A Microplate Spectrophotometer (Biotek, USA) was used to determine the absorbance at the wavelength of 450 nm. The assay was performed in triplicate. The proliferation inhibition rate is calculated based on the formula: inhibition rate = (1-Absorbance of treated sample/Absorbance of control sample) × 100%.

### HDAC colorimetric activity assay

Effects of VA on HDAC activity in both PC‐3 and DU145 cells were evaluated in triplicate using the Colorimetric HDAC Activity Assay Kit (BioVison, USA). Cells were treated with either VA or the same volume of ddH2O (NC group). Absorbance was assessed by microplate spectrophotometer (Biotek, Winooski, USA) at the wavelength of 405 nm. HDAC activity was presented as the relative O.D. value per μg protein sample.

### 3D spheroid formation assay

Hanging drop method was applied for cellular spheroid formation which has been described in our previous study [10]. Approximately 500 PC-3 and DU145 cells were added in 30 mL of solution, which contained Corning Matrigel Matrix High Concentration (HC) (Phenol-Red-free) and complete culture medium. VA (treatment group) or an equal amount of ddH_2_O (NC group) was added into the liquid drops after 24-h incubation. The imaging was taken at 0 h, 48 h, and 96 h of drug treatment. The cross-section area was used to determine the 3D volume, which was calculated by ImageJ (version 1.52a; National Institutes of Health, USA). The cross-section area inhibition rate = (1-cross-section area of treated sample/cross-section area control sample [NC]) × 100%.

### RNA extraction and quantitative RT-PCR

PC-3 and DU145 cells cultured in either 2D or 3D systems, interfered with VA or ddH_2_O(NC) for 48 h, were used for total RNA extraction using the RNeasy mini kit (Qiagen, Germany) following the manufacturer’s instructions. Epoch microplate spectrophotometer (Biotek, USA) was applied to determine the concentration and purity of total RNA. Reverse transcription was then conducted using AffinityScript multi temperature cDNA synthesis kit (Agilent technologies, CA, USA). The expression of *E2F1* and *E2F3* genes was determined using the SYBR Green PCR Kit (Qiagen, Germany) on a 7500 Fast Real-time PCR System (Life Technologies, USA). All the primer sequences used in this study and qPCR conditions were described in our previous study [10]. Each sample was analyzed in triplicate. The dissociation curve was run after the PCR amplification in each assay. The relative expression levels of a target gene mRNA between the treatment and control groups are expressed as a fold change relative to *GAPDH* using the 2^−ΔΔCt^ method.

### Western blotting

Cell lysates were loaded in a 10% polyacrylamide gel for electrophoresis, followed by nitrocellulose membrane transference based on the standard Western blot assay protocol. 5% non-fat milk in the PBS for blocking was incubated at 37 °C for 1 h. A corresponding antibody against a specific protein (anti-HDAC3 antibody was obtained from Proteintech, USA) was added for incubation at 4 °C overnight and followed by the detection with an appropriate horseradish peroxidase conjugated secondary antibody (1/1000, Cell Signaling Technology, USA) at 37 °C for 1 h, as described in our previous study [12]. After the final PBS washing, signal was developed by ECL detection system and relative photographic density was quantitated by a gel documentation and analysis system (Alpha Imager 2000, Alpha Innotech Corporation, USA).

### Caspase-3 activity assay

The caspase-3 activity assay was conducted in both PC-3 and DU145 cells cultured in either 2D or 3D culture systems as described in our previous study [10]. Cells treated with either 100 μM VA (72 h treatment) or the same amount of ddH_2_O (NC) were regarded as induced apoptosis groups (72 h after treatment). As for inhibited apoptosis groups, cells were added with 3-mL Z-VADFMK inhibitor at 72 h. Protein concentration was determined by the bicinchoninic acid (BCA) protein assay (Thermo Fisher Scientific, USA) and the p-Nitroaniline (pNA). Calibration Curve was also made by colorimetric assay system. Eventually, the CASP3 SA (caspase-3 specific activity) is calculated using the following formula: SA = (pmol pNA liberated per hour)/mg protein.

### Prostate tumor-bearing mice modeling

SPF level, 6-week male nude mice were purchased from Beijing HFK Bioscience Company (Beijing, China). Experimental protocols were approved by the Beijing University of TCM Institutional Animal care and Use Committee (No.104195489042). For each tumor transplantation, fresh prepared 5 × 10^6^ PC-3 cells (mixed in 50 μL complete F-12 K Medium and 50 μL Corning® Matrigel® Matrix High Concentration, Phenol-Red, and LDEV-free) were subcutaneously injected into right axilla of mice. Mice were then randomly divided into three groups: VA group (*N* = 4), NC group (*N* = 4), and CDDP group (*N* = 4). 50 mg/kg VA and the same volume of ddH_2_O were given to VA group and NC group by gavage, every 2 days, respectively. 1 mg/kg Cisplatin (CDDP, cis-Diaminodichloroplatinum) (InvivoChem, USA) was given to each mice in CDDP group by intraperitoneal injection, every two days. VA + CDDP group (VA combined with CDDP) was given same amount agents with VA and CDDP group. After 14 days of intervention, all nude mice were sacrificed, and the tumor weight were evaluated to calculate the tumor inhibition rate. Tumor inhibition rate (TIR) (%) = (1-average tumor weight in the treatment group/average tumor weight in the control group) × 100%.

### Detecting the apoptosis rate and cell cycle by flow cytometry

The apoptosis rate and cell cycle were analyzed using flow cytometry [10]. After the tumor tissue was taken, a single-cell suspension was made using a homogenizer, followed by centrifugation at 1000 r·min^−1^ for 5 min. Cells were stained according to Annexin V-FITC/PI kit instructions, and the results were analyzed by flow cytometry (model FACSVia, BD company). Each group of experiments was repeated three times, and CellQuest 3.0 software was used to analyze the apoptosis rate.

### Statistical analysis

For each assay, at least 3 independent experiments were performed in triplicate. Means and standard deviations (SD) were calculated, and data are presented as mean ± SD. Differences between groups were determined using a generalized linear model with post hoc Tukey test for the correction of multiple comparisons. Inhibition rate = (1-Absorbance of treated sample/Absorbance of control sample) × 100%. Statistical significance was considered when *P* < 0.05 (two-sided). All statistics and figures were generated using GraphPad Prism 9.0 software (www.graphpad.com) or R packages (version 3.6.1).

## Results

### VA as a potential HDACs inhibitor

**Fig. 1:**
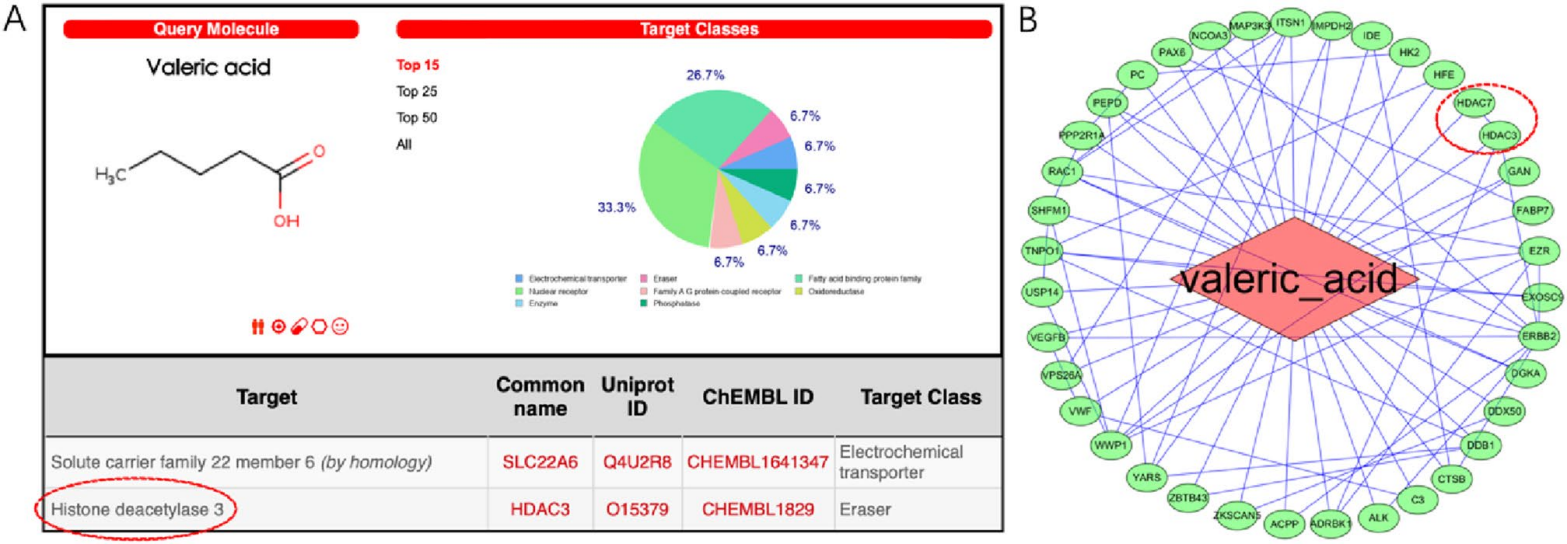
Valeric Acid targets the HDACs. HDAC3 and 7 were predicted to be the potential targets of VA, respectively, by Swiss target prediction assay (**A**) and PPI network (**B**)

As shown in Fig. 1, HDAC3 ranked second, as a predicted potential target of VA in Swiss target prediction assay. The frequencies of target class were 53% for Enzyme, 40% for Unclassified, and 7% for protease, respectively (Fig. 1A). As shown in PPI network (Fig. 1B), 37 potential targets of VA were obtained by PharmMapper database. Among the network of predicted protein–protein interaction relationships, HDAC3 and HDAC7 were also the predicted targets of VA.

### VA was predicted to affect HDAC activity and prostate cancer

**Fig. 2:**
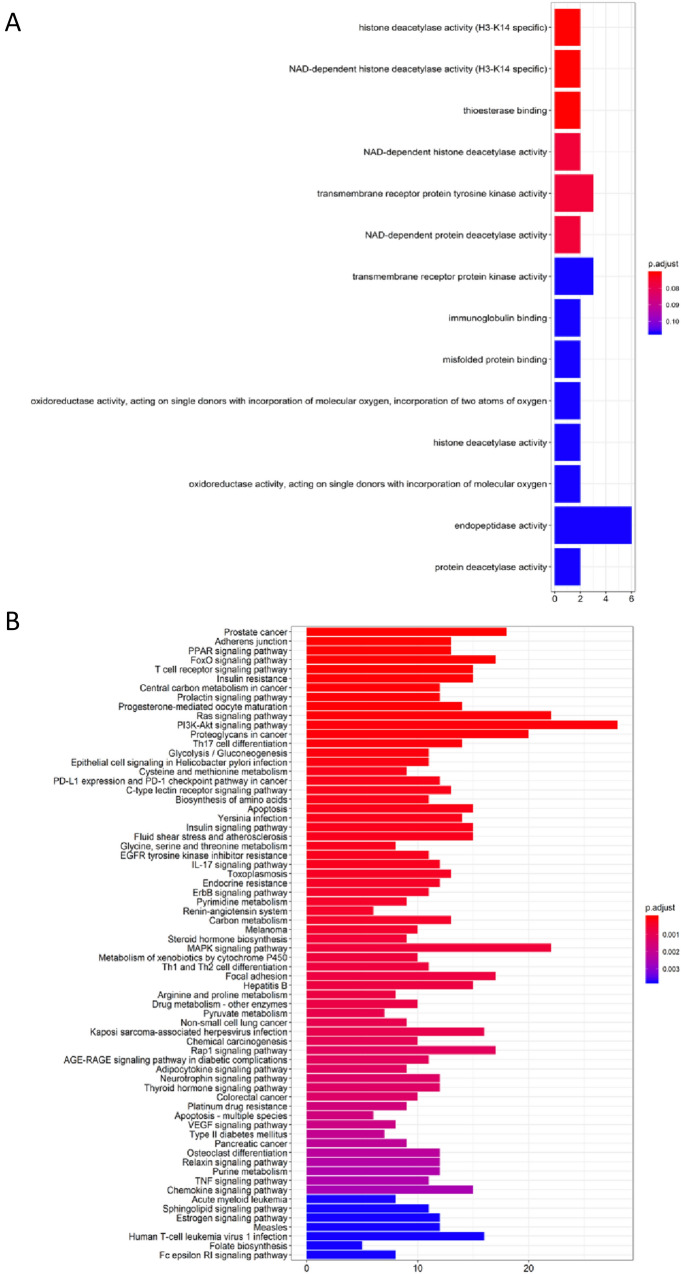
VA has been predicted to impact HDAC activity and prostate cancer. “Histone deacetylase activity (H3-K14 specific)” and “histone deacetylase activity” ranked at the first (*P* < 0.05) and 11th place (*P* > 0.05), respectively. Both of them have been predicted to be regulated by VA (**A**). The KEGG pathways analysis (**B**) displayed the potential diseases or pathways that might be affected by VA. The top predicted result is prostate cancer

As shown in Fig. 2, GO function enrichment analysis displayed the top 14 predicted functional targets of VA. “Histone deacetylase activity (H3-K14 specific)” and “histone deacetylase activity” ranked at the first (*P* < 0.05) and 11th place (*P* > 0.05) (Fig. 2A), respectively. Both of them have been predicted to be regulated by VA. The KEGG pathway analysis (Fig. 2B) displayed the potential diseases or pathways that might be effected by VA. The top five pathways were Prostate cancer, Adherens junction, PPAR signaling pathway, FoxO signaling pathway, and T-cell receptor signaling pathway, indicating that these pathways might underlie the pharmacological effects of VA.

### Downregulation of *HDAC3* expression by VA

**Fig. 3:**
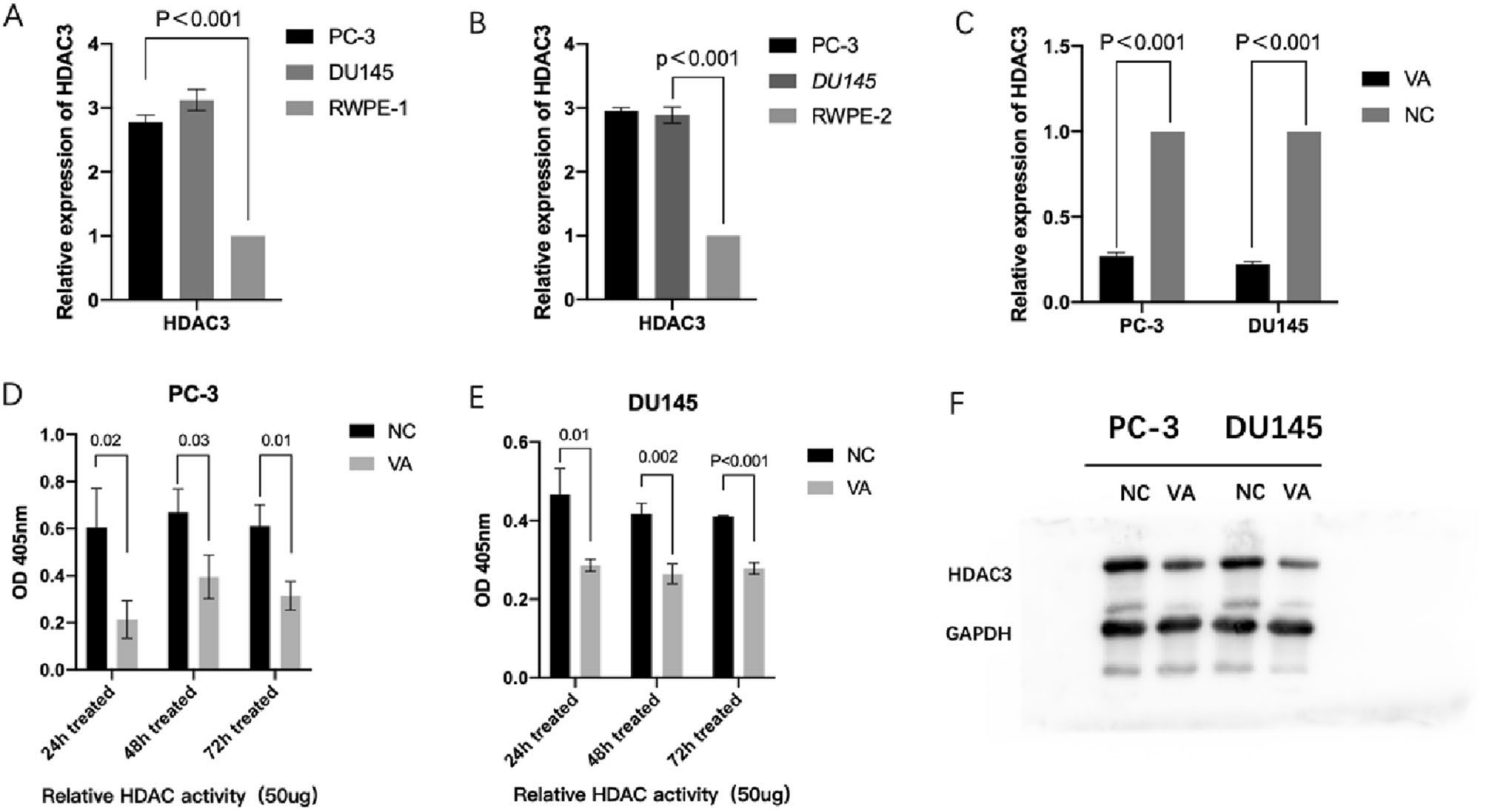
VA suppresses the expression of HDAC3. In both PC-3 and DU145 cells, after 48-h treatment of VA, and the expression of HDAC3 was reduced significantly, compared with NC group (*P* < 0.05) (**A**–**E**). Meanwhile, in both PC-3 and DU145 cells, after 48-h treatment of VA, the expression of HDAC3 protein was reduced, compared with NC group (**F**)

As shown in Fig. 3, RT-qPCR results displayed that both prostate cancer cell lines had higher *HDAC3* expression compared to two normal prostate cell lines, respectively (Fig. 3A and B). After the treatment, in PC-3, the relative expression levels of *HDAC3* in the VA groups was 0.27 ± 0.02-fold of the control group (*P* < 0.001). The relative expression levels of *HDAC3* in DU145 was 0.224 ± 0.015-fold of the control (*P* < 0.001) (Fig. 3C). In addition, the HDAC activity assay results showed that VA significantly decreased HDAC activity in both tested prostate cancer cells at 24 h, 48 h, and 72 h after intervention. For example, in PC-3 cells, the normalized HDAC activity of the NC group (0.21 ± 0.092) was significantly higher than that of VA-treated group (0.61 ± 0.16) at 24 h (*P* < 0.05) and the trend remained at 72 h (Fig. 3D). Similar trends were also observed in cell line DU145 (Fig. 3E). It has also been shown that the expression of HDAC3 protein has been suppressed by the intervention of VA, in either PC-3 or DU145 cell line, compared with NC group, respectively (Fig. 3F).

### VA suppressed proliferation of prostate cancer cell in vitro

**Fig. 4:**
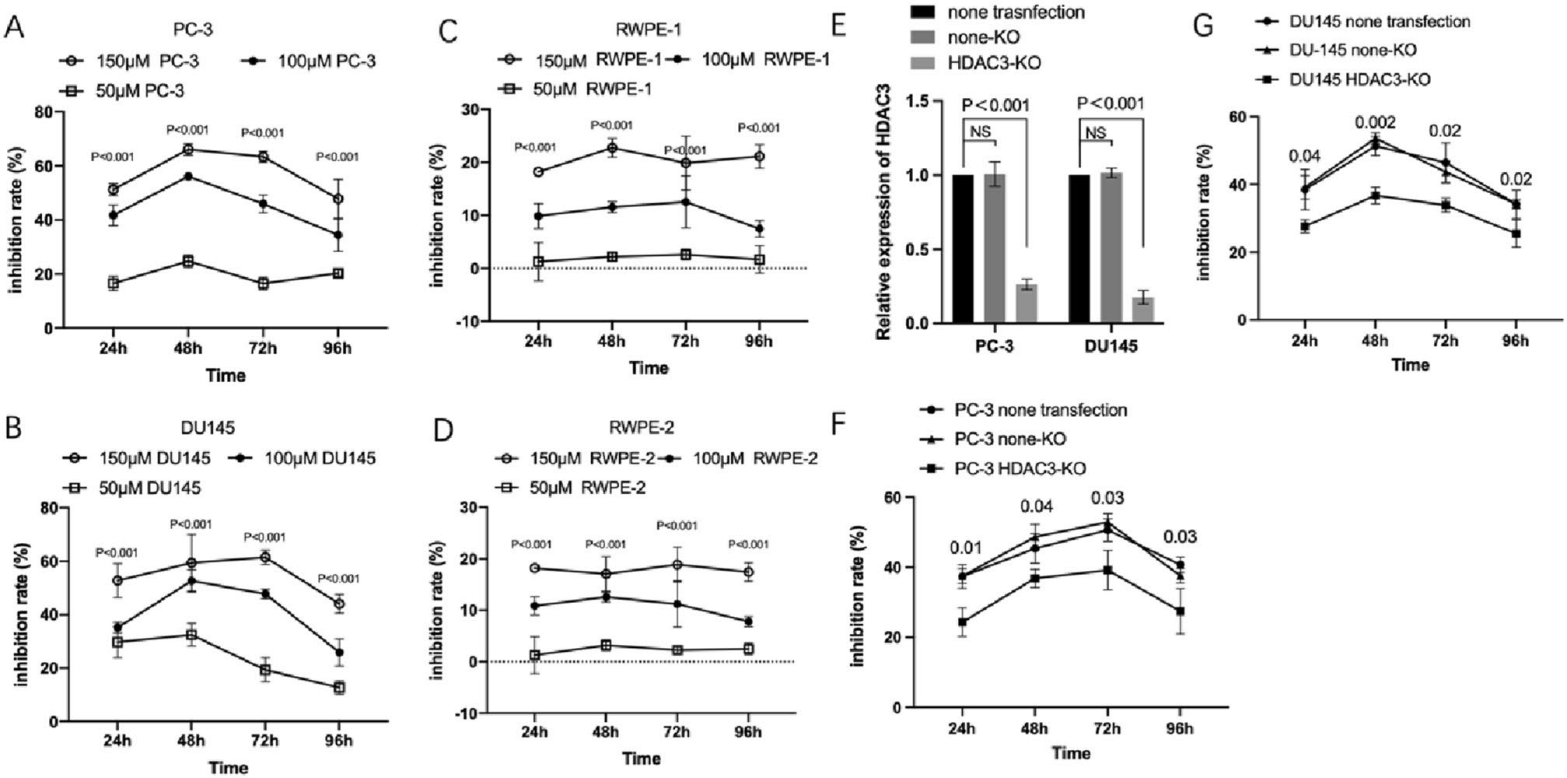
Effect of VA on proliferation of prostate cancer cell lines. VA has anti-proliferative effect on prostate cancer cells while being low toxic to normal cells and such effect could be reduced by *HDAC3* gene knockout. The inhibition rates of PC‐3 in the presence of 100 μM VA ranged from 34.42 ± 5.9% to 56.08 ± 1.28% during 24 h to 96 h and displayed significantly higher inhibition rates when compared to the 50-μM VA group with the range from 16.52 ± 2.32% to 24.76 ± 2.32% (*P* < 0.001, from 24 to 96 h) (**A**). Similar trends have been displayed in DU145 (**B**). Moreover, for RWPE-1 and RWPE-2, the inhibition rates of 50 μM VA group were significantly lower when compared to 100 μM group, respectively (**C** and **D**). The relative HDAC3 expression was significantly decreased in HDAC3-KO group than none transfection group in both PC-3 and DU145 cell lines (**E**). The inhibition rates were significantly decreased in CAXII-KO group compared to both non-transfection group and none-KO group, at 24, 48, 72, and 96 h, respectively, in PC-3 and DU145 cell lines (**F** and **G**)

To explore the biological effect of VA on both prostate cancer cell and prostate normal cells in vitro, cancer cell lines of PC‐3 and DU145 and normal cell lines of RWPE-1 and RWPE-2 were treated with either VA or ddH2O, respectively. The inhibition rates of PC‐3 in the presence of 100 μM VA ranged from 34.42 ± 5.9% to 56.08 ± 1.28% during 24 h to 96 h and displayed significantly higher inhibition rates when compared to the 50-μM VA group with the range from 16.52 ± 2.32% to 24.76 ± 2.32% (*P* < 0.001, from 24 to 96 h) (Fig. 4A). The inhibition rate of DU145 in the 100-μM VA group ranged from 25.82 ± 5.07 to 52.64 ± 4.17 and also displayed significant differences to 50 μM VA group from 24 to 96 h (*P* < 0.001, from 24 to 96 h, respectively) (Fig. 4B). Moreover, for RWPE-1 and RWPE-2, the inhibition rates of 50 μM VA group were significantly lower when compared to 100 μM group, respectively (Fig. 4C and D).

### Expression of HDAC3 effects the anti-proliferative ability of VA

As shown in Fig. 4E, both PC-3 and DU145 cells, the relative expression level of HDAC3 in HDAC3-KO (HDAC3 knockout) group was significantly deceased than that in none-KO (none knockout) and none transfection groups (*P* < 0.001), respectively. To evaluate the effect of *HDAC3* knockout on the anti-proliferative effect of VA on prostate cancer, inhibition rates of VA on PC‐3 and DU145 cells with or without *HDAC3* knockout have been examined, respectively. For details, at 24 h, in PC-3 cells, the inhibition rate of HDAC3-KO group (24.34 ± 4.09%) was significantly lower than that of none transfection group (37.41 ± 3.37%) (*P* < 0.05) and none-KO group (37.52 ± 1.75%) (*P* < 0.05), respectively. Similar trends were observed at other time points and also in DU145 cell (Fig. 4F and G).

### VA inhibited 3D spheroid formation, increased caspase‑3 activity, and suppressed the expression of *E2F1*/*E2F3*

**Fig. 5:**
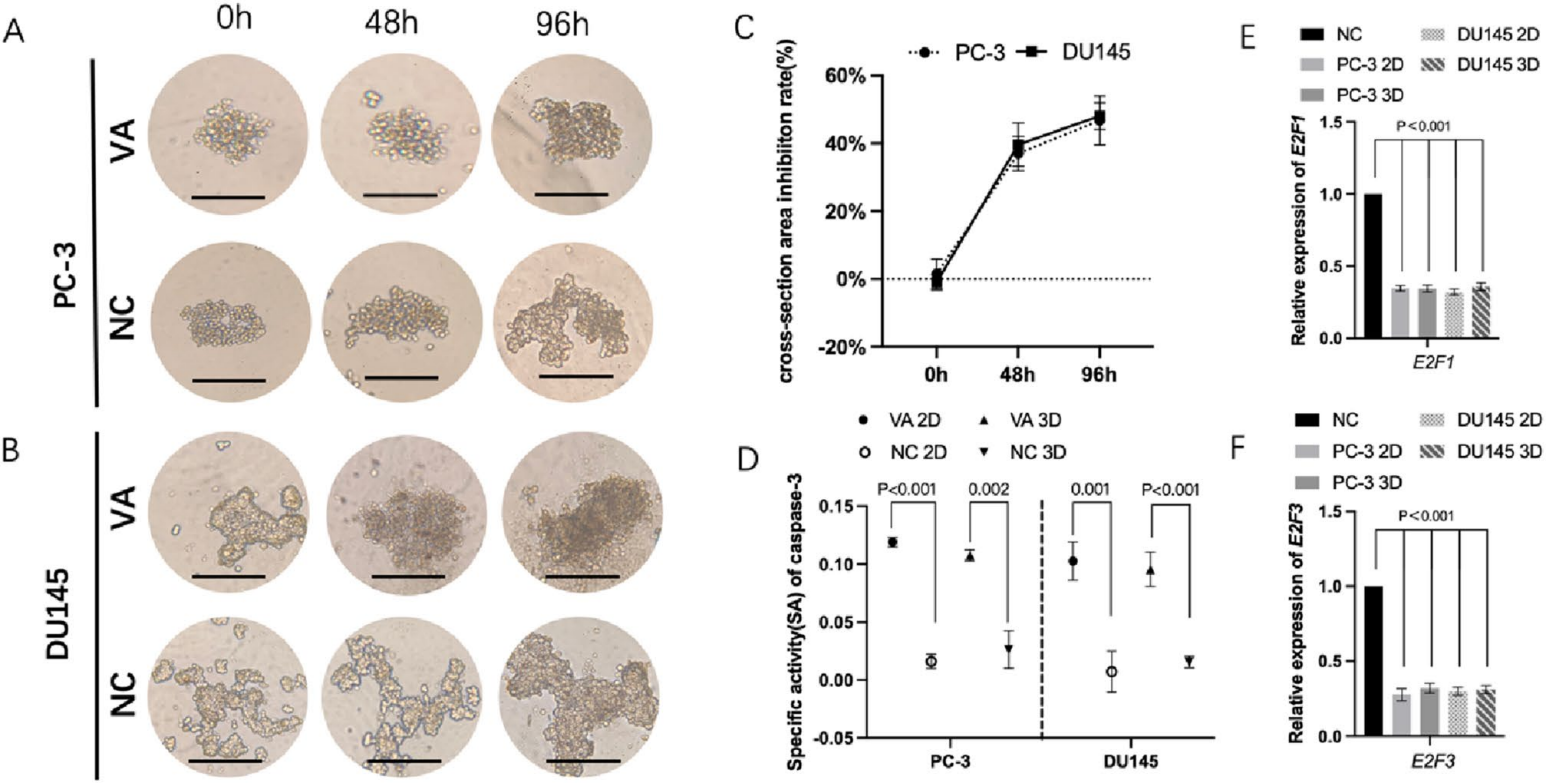
The representative 3D spheroid models of PC-3 (**A**) and DU145 (**B**) cells were treated by VA and NC, respectively. The cross-section area inhibition rates eventually climbed to 46.77 ± 19.62% and 48.07 ± 19.72% at 96 h for PC-3 and DU145 cells, respectively (**C**). The CASP3 SA (caspase-3 specific activity) has shown that VA evaluated the caspase-3 activity in both PC-3 and DU145 cells cultured in either 2D or 3D system, compared to respective NC (*P* < 0.05) (**D**). Relative expression of *E2F1* and *E2F3* in VA group was significantly lower that of NC, respectively (*P* < 0.001) (**E, F**), *NC* negative control

The dynamic changes of 3D spheroid formation of PC-3 and DU145 cells are shown in Fig. 5A and B. For PC-3 cell, moreover, the cross-section area inhibition rate raised from 1.56 ± 3.15% at beginning to 46.77 ± 19.62% at 96 h. As for DU145 cell, the inhibition rate has climbed from − 0.93 ± 2.34% to 48.07 ± 19.72% at 96 h (Fig. 5C). The caspase-3 SA in the VA group was significantly higher compared to the control group in both PC-3 and DU145 cells cultured in 2D and 3D, respectively (*P* < 0.05). For details, in 2D-cultured system PC-3 cells, the SA in VA group (0.12 ± 0.003) was significant higher than that in NC group (0.02 ± 0.005, *P* < 0.001). As for PC-3 cells in 3D-cultured system, the SA in VA group (0.11 ± 0.004) was also significantly higher than that in NC group (0.02 ± 0.013, *P* = 0.002). Similar trend has also been detected in DU145 cells (Fig. 5D–F).

Moreover, RT-qPCR results showed that, in PC-3, at 48 h, the relative expression levels of *E2F1* in the *VA* group were 0.34 ± 0.02(2D) and 0.34 ± 0.02(3D) folds of the each control groups, respectively (*P* < 0.001), and 0.32 ± 0.01(2D) and 0.36 ± 0.02(3D) folds in DU145 cells, respectively (*P* < 0.001). Similar trends were found in *E2F3* (Fig. 5G).

### Inhibition of prostate cancer by VA in vivo

**Fig. 6:**
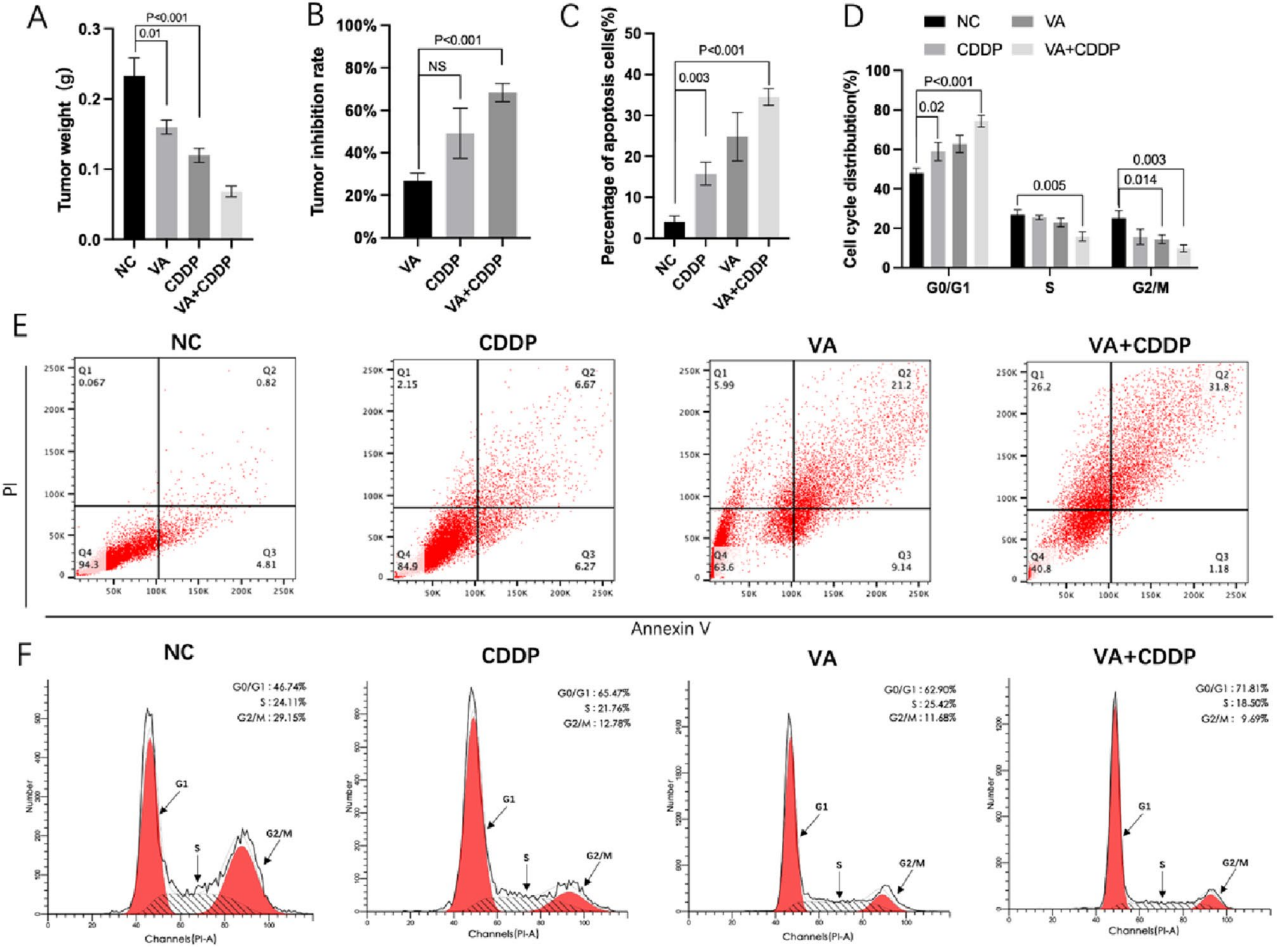
Effects of VA on PC-3 cell in vivo. Treatment of single VA, single CDDP, and VA + CDDP significantly reduced the tumor weight of prostate cancer-bearing mice (**A**). Tumor inhibition rate (TIR) VA + CDDP group was significantly higher than that in VA and CDDP group (*P* < 0.05), respectively **B** VA induced the cell apoptosis and arrested the cell cycle of PC-3 cells. Flow cytometry analysis found that either VA or CDDP can significantly increase the apoptosis rate compared to NC group (*P* < 0.05), respectively (**C**, **E**). Treatments significantly increased the proportion of G0/G1 phase cells and significantly decreased the proportion of S phase and G2/M cells (**D**, **F**)

As shown in Fig. 6A, the tumor weight was significantly lower in VA and CDDP groups, compared with NC group, respectively (*P* < 0.05). Moreover, tumor weight of VA + CDDP group was also significantly lower than that of either VA or CDDP group, respectively (*P* < 0.05). As for tumor inhibition rate (TIR), even though no significant difference has been observed between VA (30.73 ± 8.24%) and CDDP group (47.87 ± 8%) (*P* = 0.102), the TIR of VA + CDDP group (70.33 ± 4.67%) was significantly higher than that of VA (*P* = 0.004) and CDDP group (*P* = 0.027), respectively (Fig. 6B).

As displayed in Fig. 6C and E, the apoptosis rate in VA (24.82 ± 4.79) and CDDP group (15.78 ± 2.29) was also significantly higher than that in NC group (4.02 ± 1.18) (*P* < 0.05), respectively. Furthermore, apoptosis rate of CDDP + VA group (34.5 ± 1.7) was also higher than that of VA and CDDP group, respectively (*P* < 0.05).

As shown in Fig. 6D and F, both CDDP and VA alone can cause G0/G1 phase arrest of PC-3 cells compared to NC group, respectively (*P* < 0.05). However, the proportion of cell treated by VA + CDDP at G0/G1 phase was significantly higher than that of VA and CDDP group, respectively (*P* < 0.05). In contrast, the proportion of cell treated by VA + CDDP at G2/M phase was significantly lower than that of NC, VA, and CDDP group, respectively (*P* < 0.05).

## Discussion

Histone deacetylases (HDACs) are frequently overexpressed in a broad range of cancer types. Up to now, four classes of HDACs containing 18 isozymes are known. Class I HDAC members, including HDAC 1, 2, 3, and 8, are widely localized in the nuclei of all tissues. In class I HDAC members, HDAC3 plays a very important role in DNA damage control and genomic stability and modifying cellular epigenetic programming, therefore affecting cell proliferation and survival. Moreover, HDAC3 inhibitor has been found to hold strong proapoptosis ability and might suppress cancer stemness [5–7, 10]. Some evidence has also indicated that HDACi might increase the therapeutic effect of chemotherapy [13]. HDAC inhibitors, e.g., SAHA for cutaneous T-lymphocyte tumors, Mocetinostat for bladder cancer, and Panobinostat for multiple myeloma, were approved by the U.S. FDA [14, 15]. Meanwhile, it has been found that overexpression of HDAC3 has been found in a positive correlation with the proliferation, development, and poor prognosis of prostate cancer [6]. Based on what we found previously that VA is a potential HDAC inhibitor which holds multiple anti-cancer effects for liver and breast cancer [10, 11], the effects of VA on prostate cancer seem also more intriguing.

Both *E2F1* and *E2F3*, as oncogenes, are involved in the regulation of many biological properties, such as cancer stemness and apoptosis. *E2F1* can affect cell growth factors by regulating NF-κB, thereby promoting tumor proliferation and anti-apoptosis. *E2F1* can also inhibit the transcription of ICAM-1 leading to immune escape. Overexpression of miR-34a and miR-214 was also found to inhibit the proliferation of hepatoma cells by downregulating the expression of *E2F3* [16]. Generally, *E2F1/E2F3*, as an important node molecule of cell signaling network, participate in the transcriptional regulation of many important genes through dialogue with other signaling pathways, including NF-κB, and are tumor therapy targets [17, 18].

Caspase-3, as a member of the aspartase family, is an executive factor that acts on Caspase-8 and 9 in mediating apoptosis. Recently, it has been found that it may affect physiological processes such as stemness and autophagy of tumor cells and play an important role in tumor development and prognosis [17]. CASP3 expression is positively correlated with the level of tumor cell apoptosis [18]. In addition, Caspase-3 can also mediate other important physiological functions, such as the regulation of tumor cell pyroptosis by mediating GSDM [19]. More importantly, previous study has proven that *E2F1*/*E2F3*/Caspase-3 axis is an effective mechanism in cancer suppression which not only can advance apoptosis level in cancer cell but also inhibit stemness, invasion, migration, etc. [16, 17]. VA, as a novel HDAC inhibitor, was shown regulating this axis in liver cancer [10].

In this study, Network pharmacology research methods (Network pharmacology research and Swiss prediction tool) have been applied to predict the role and function of VA in prostate cancer based on its components and structure [20–23]. As shown in our results, HDAC family members, including HDAC3, 6, and 7, have all been predicted to be the potential targets of VA by Swiss target prediction assay and PPI network, and HDAC3 is the top one in Swiss target prediction. Furthermore, in GO function enrichment analysis, “histone deacetylase activity” has been predicted to be regulated by VA (*P* < 0.05). Meanwhile, VA has also been displayed to make effects on “prostate cancer” (*P* < 0.001) which has preliminarily revealed the close relationship between VA, prostate cancer, and HDAC. Therefore, we believed that VA still occupies the property of HDACi in prostate cancer treatment and can inhibit the development of prostate cancer.

In line with these theoretic results, our in vitro cell line results have further validated the inhibitive effect of VA on expression of HDAC3 in prostate cancer cells and HDAC3 activity levels. The results also showed that VA possessed the anti-proliferative ability in prostate cancer cells and such ability was partly exerted by inhibiting HDAC3. Meanwhile, VA can also suppress the spheroid formation ability of selected prostate cancer cells. The anti-cancer effect of VA was further tested in vivo*,* and we found that the tumor weight was significantly lower in VA compared with NC group (*P* < 0.05). Oppositely, the apoptosis level evaluated by Flow cytometry analysis in VA group was also significantly higher than that in NC group (*P* < 0.05). Interestingly, as shown in Fig. 6A, tumor weight of VA + CDDP group has been found to be significantly lower than that of either VA or CDDP group, respectively (*P* < 0.05). According to that, the TIR of VA + CDDP group was also found to be significantly higher than that of VA and CDDP group, respectively. Same trends were also detected in cell apoptosis and cell cycle analyses, which suggested that the VA increased apoptosis and the arrest of cell cycle, further enhancing the anti-cancer ability of CDDP.

Therefore, we further explored relevant mechanisms. Our RT-PCR assay showed that the expression of *E2F1/E2F3* have been downregulated by VA in either 2D- or 3D-cultured systems. Under the same conditions, the Caspase-3 activity has been improved by VA, which indicates that *E2F1*/*E2F3*/Caspase-3 axis may be one of the anti-cancer mechanism of VA.

Limitations exist in this study. We preliminarily revealed the anti-cancer effects of VA on prostate cancer in vitro and in vivo as an HDACi. Immune-deficient nude mouse models limit us to investigate the effect of HDACi on immune response. However, more preclinical evidence should be added to comprehensively evaluate the application potential of VA on prostate cancer. For instant, we plan to use Patient-derived tumor xenograft (PDX) model to conduct animal study instead of cell-derived xenograft (CDX) model in our future study. Moreover, we successfully cultured selective cells in 3D-cultured system in this study, which allow us to try Patient-derived organoids (PDOs) formation assay in our next research. In addition, PDX model in humanized mice will be very interesting, which will allow us to investigate the involvement of immune system in HDACi against the tumor.

In summary, this is the first study to display that VA possesses the potential to regulate HDACs in prostate cancer cells. More specifically, it inhibits the expression of HDAC3 therefore suppressing the proliferation of prostate cancer cells. Meanwhile, regulating *E2F1*/*E2F3*/Caspase-3 axis may be one way that VA blocks stemness and advances apoptosis in prostate cancer cells. These findings suggest the potential of VA as a novel chemosensitizer. Based on the fact that HDAC inhibitors have been applied as an anti-cancer agent, suggesting that VA could be a potential novel HDAC inhibitor in prostate cancer treatment. Further investigation of VA is also warranted to explore its therapeutic effect and mechanisms.
